# Unveiling Novel Mechanism of CIDEB in Fatty Acid Synthesis Through ChIP-Seq and Functional Analysis in Dairy Goat

**DOI:** 10.3390/ijms252011318

**Published:** 2024-10-21

**Authors:** Qiuya He, Weiwei Yao, Jiao Wu, Yingying Xia, Yuanmiao Lei, Jun Luo

**Affiliations:** Shaanxi Key Laboratory of Molecular Biology for Agriculture, College of Animal Science and Technology, Northwest A&F University, Yangling, Xianyang 712100, China; heqiuya@nwafu.edu.cn (Q.H.); yaoweiwei@nwafu.edu.cn (W.Y.); 15229238680@163.com (J.W.); xiayingying0604@163.com (Y.X.); lemon.lym@outlook.com (Y.L.)

**Keywords:** CIDEB, milk fatty acids, transcriptional regulation, XBP1, goat mammary epithelial cells

## Abstract

Goat milk is abundant in nutrients, particularly in milk fats, which confer health benefits to humans. Exploring the regulatory mechanism of fatty acid synthesis is highly important to understand milk composition manipulation. In this study, we used chromatin immunoprecipitation sequencing (ChIP-seq) on goat mammary glands at different lactation stages which revealed a novel lactation regulatory factor: cell death-inducing DFFA-like effector B (CIDEB). RT-qPCR results revealed that CIDEB was significantly upregulated during lactation in dairy goats. CIDEB overexpression significantly increased the expression levels of genes involved in fatty acid synthesis (*ACACA*, *SCD1*, *p* < 0.05; *ELOVL6*, *p* < 0.01), lipid droplet formation (*XDH*, *p* < 0.05), and triacylglycerol (TAG) synthesis (*DGAT1*, *p* < 0.05; *GPAM*, *p* < 0.01) in goat mammary epithelial cells (GMECs). The contents of lipid droplets, TAG, and cholesterol were increased (*p* < 0.05) in CIDEB-overexpressing GMECs, and knockdown of CIDEB led to the opposite results. In addition, CIDEB knockdown significantly decreased the proportion of C16:0 and total C18:2. Luciferase reporter assays indicated that X-box binding protein 1 (XBP1) promoted CIDEB transcription via XBP1 binding sites located in the CIDEB promoter. Furthermore, CIDEB knockdown attenuated the stimulatory effect of XBP1 on lipid droplet accumulation. Collectively, these findings elucidate the critical regulatory roles of CIDEB in milk fat synthesis, thus providing new insights into improving the quality of goat milk.

## 1. Introduction

Dairy goats play a crucial role in China’s dairy industry and hold a top position globally in terms of production volume and population. Goat milk and its products are favored by consumers due to its high nutritional value, easy digestibility, and low allergy potential [1]. Our recent findings demonstrate that dietary intervention with goat milk can reduce blood glucose levels in type 2 diabetic mice and contribute to the restoration of pancreatic function [2]. Additionally, goat milk has been reported to have potential preventive effects on conditions such as atherosclerosis and liver cirrhosis [3]. Furthermore, the flavor of goat milk is closely associated with a higher content of short- and medium-chain fatty acids such as caproic acid, caprylic acid, and capric acid in goat milk fat [4]. Thus, exploring the regulatory mechanisms of fatty acid metabolism in goat milk is of paramount scientific significance for understanding the genesis of goat milk nutritional value.

Cell death-inducing DFFA-like effector b (CIDEB) is associated with both the endoplasmic reticulum and lipid droplets, and facilitate their fusion and regulate their size [5]. Recent studies have found that CIDEB can promote fatty acid synthesis and adipocyte formation and participate in triglyceride synthesis and storage in the liver [6,7].

Additionally, CIDEB is believed to play a regulatory role in the development of metabolic disorders such as insulin resistance and obesity [8]. Deletion of CIDEB in the mouse liver reduces cholesterol biosynthesis rates and alters plasma cholesterol levels by decreasing the expression of the SREBP cleavage-activating protein (SCAP) [9]. Moreover, *CIDEB* knockout also diminishes SREBP signaling in the liver, thereby regulating lipid metabolism [10]. Interestingly, the morphology of lipid droplets is smaller in CIDEB deficient mice, and increased fatty acid oxidation is observed in the liver [8]. Furthermore, CIDEB enhances the hepatic lipid storage capacity by promoting the fusion and expansion of lipid droplets [11]. These findings highlight the importance of CIDEB as a lipid droplet-associated protein that regulates lipid metabolism.

X-box binding protein 1 (XBP1) is a transcription factor that plays a crucial role in various cellular processes, including lipid metabolism [12]. Moreover, XBP1 activates gene expression involved in fatty acid synthesis, such as fatty acid synthase (FASN) and stearoyl-CoA desaturase 1 (SCD1) [13], leading to increased fatty acid production and lipid accumulation. Additionally, activated *XBP1* can directly bind to the promoter region of peroxisome proliferator-activated receptor gamma (PPARG), enhancing its transcriptional activity and thus promoting lipid droplet accumulation [14]. Previous study has revealed that *XBP1* regulates *SREBP2* transcription, and enhances de novo cholesterol biosynthesis by inhibiting SREBP2 protein ubiquitination [15]. Moreover, dysregulation of XBP1 is associated with metabolic disorders, including obesity, insulin resistance, and non-alcoholic fatty liver disease [16,17]. It is plausible that CIDEB may modulate fatty acid synthesis in GMECs through XBP1-mediated regulation of its own expression.

The objective of this study aimed to explore the regulatory mechanisms of CIDEB in goat milk fatty acid metabolism at the lactation stage. CIDEB positively regulated fatty acid synthesis and lipid droplet accumulation in goat mammary epithelial cells (GMECs). Additionally, XBP1 was identified as a key regulator of CIDEB activity because it directly binds to the promoter region. Understanding the intricate interplay among CIDEB, XBP1, and fatty acid synthesis provides crucial insight into the molecular mechanisms underlying milk lipid metabolism.

## 2. Results

### 2.1. Identification of CIDEB Through Chromatin Immunoprecipitation Sequencing (ChIP-Seq) in Dairy Goats

**Figure 1:**
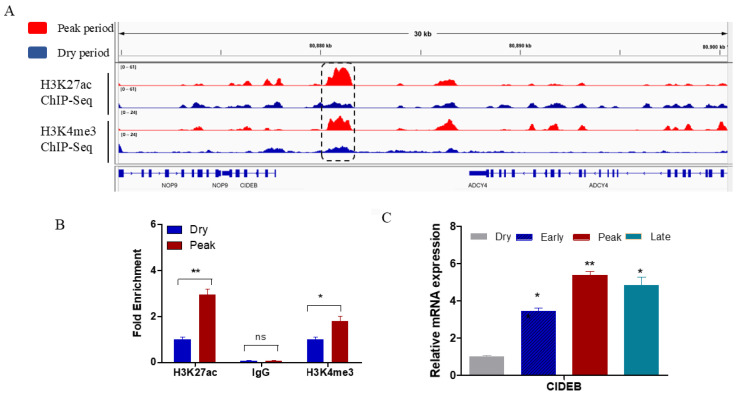
Identification of active gene for fatty acid synthesis via ChIP-seq assay of H3K27ac and H3K4me3. (**A**) H3K27ac and H3K4me3 peaks were both located in the CIDEB promoter region and upregulated during the peak lactation stage. CIDEB promoter is highlighted in black boxes. (**B**) ChIP-qPCR analysis of H3K27ac and H3K4me3 enrichment in the CIDEB promoter region between the peak lactation group and dry period group. (**C**) Different expression levels of the CIDEB gene during dairy goat lactation. The values represent the mean ± standard error of the mean (SEM) for three biological replicates: ns, *p* > 0.05, *, *p* < 0.05, **p < 0.01.

Transcriptional activation of gene expression is usually achieved through cis-regulatory modules such as enhancers and promoters, which function to determine when and where transcription occurs [18]. H3K27ac and H3K4me3 are considered markers of active enhancers and promoters [19,20]. To identify genome-wide active cis-regulatory modules, we assayed H3K4me3 and H3K27ac enrichment at dry and peak lactation periods in dairy goat genomes using ChIP-seq. Visualization with the Integrative Genomics Viewer (IGV) showed that the peaks of H3K4me3 and H3K27ac at the CIDEB promoter region were significantly higher during the lactation period (red peaks) compared to the dry period (blue peaks) (Figure 1A). ChIP-qPCR confirmed that the levels of the active histone marks H3K27ac and H3K4me3 on the CIDEB promoter were markedly elevated during the peak lactation period (Figure 1B). Next, we observed that CIDEB expression was highest during peak lactation than it was during the dry period (Figure 1C). Thus, we speculate that the CIDEB gene plays a crucial role during the lactation process in the goat mammary gland.

### 2.2. CIDEB Promotes Fatty Acid Synthesis and Triglyceride Content

**Figure 2:**
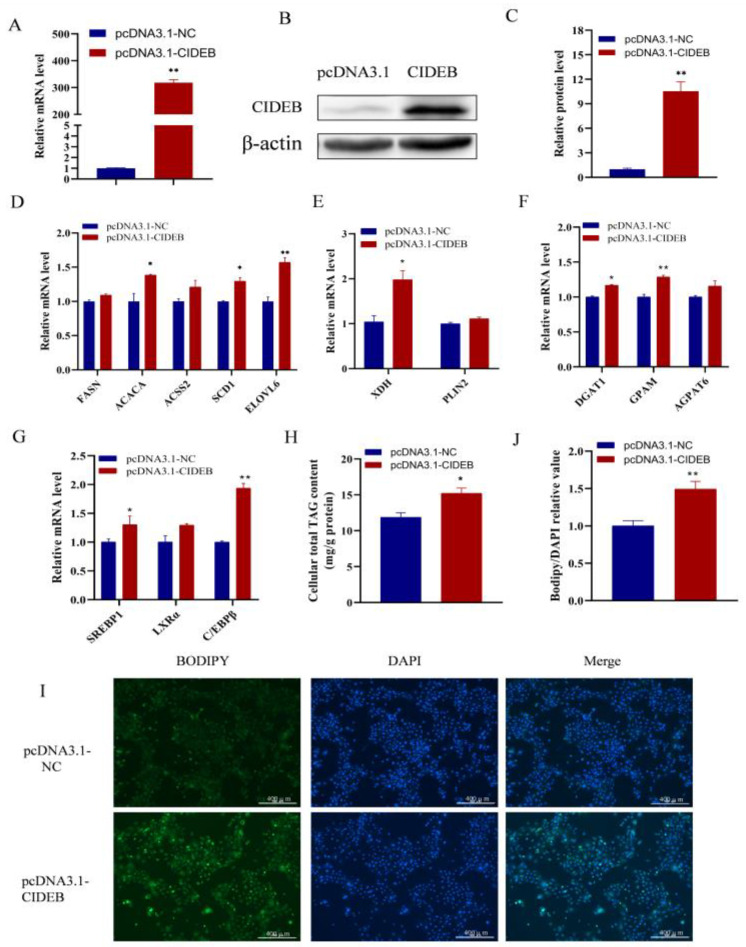
Effect of CIDEB overexpression on the expression of genes related to fatty acid metabolism in GMECs. (**A**) CIDEB mRNA and (**B**,**C**) protein levels after transfection with CIDEB-pcDNA3.1 in GMECs. (**D**–**G**) Effect of overexpression of CIDEB on the mRNA levels of genes related to (**D**) de novo synthesis of fatty acids, (**E**) lipid droplet formation, (**F**) TAG synthesis, and (**G**) transcription factors related to lipid metabolism. (**H**) CIDEB overexpression increased the intracellular TAG content. RT-qPCR data were calculated using the 2^−ΔΔCt^ method. Data represent the mean ± SEM. Statistically significant differences are indicated as follows: ** *p* < 0.01. * *p* < 0.05. (**I**,**J**) Effect of CIDEB overexpression on lipid droplet formation. GMECs were transfected with CIDEB -pcDNA3.1 and pcDNA3.1 for 48 h. Lipid droplet in GMECs were detected by BODIPY 493/503 staining. Scale bar = 400 μm.

CIDEB plays a crucial role in regulating lipid metabolism and related diseases, and it serves as a key modulator of lipid homeostasis [11]. However, studies on the involvement of CIDEB in milk lipid metabolism are limited. To explore the mechanism of CIDEB in GMECs, the CIDEB-pcDNA3.1 construct was used to induce CIDEB overexpression. RT-qPCR and western blotting results revealed that CIDEB expression was substantially upregulated after transfection of the CIDEB-pcDNA3.1 construct in GMECs (Figure 2A–C). Subsequently, we observed that *CIDEB* overexpression significantly upregulated the mRNA levels of *SCD1*, *ACACA*, and *ELOVL6* (*p* < 0.05; Figure 2D). Furthermore, the mRNA level of *XDH* (involved in lipid droplet formation), *DGAT1*, and *GPAM* (related to triglyceride (TAG) synthesis) were increased following CIDEB overexpression (Figure 2E,F). We also discovered that overexpression of *CIDEB* markedly elevated the *SREBP1* and *CEBPB* expression (Figure 2G), which are important transcription factors for fatty acid metabolism. Importantly, we observed that overexpression of CIDEB led to a significant increase in cellular TAG content (*p* < 0.05; Figure 2H) as well as the accumulation of lipid droplets, compared to the control group (Figure 2I,J). Collectively, these results indicate that overexpression of CIDEB promotes the synthesis of fatty acids and the accumulation of lipid droplet in GMECs.

### 2.3. CIDEB Deficiency Suppresses Fatty Acid Synthesis and TAG Synthesis

**Figure 3:**
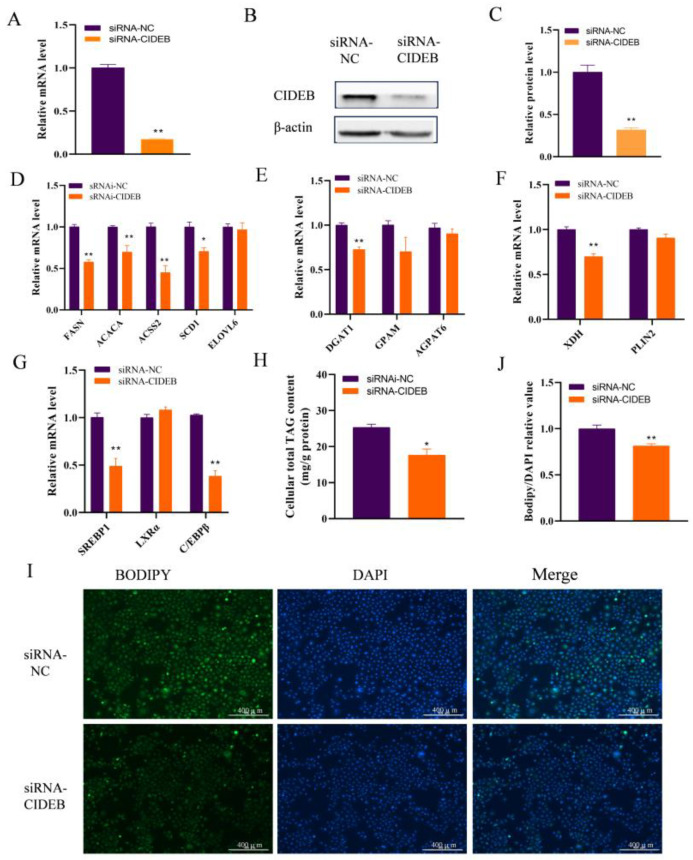
Impact of *CIDEB* knockdown on the expression of genes related to fatty acid metabolism in GMECs. (**A**) RT-qPCR analysis and (**B**,**C**) protein level of *CIDEB* after infection with siRNA- CIDEB. (**D**) *CIDEB* knockdown decreased the expression of genes related to de novo FA synthesis (*FASN*, *ACACA,* and *ACSS2*), (**E**) TAG synthesis (*DGAT1*), (**F**) lipid droplet formation, and (**G**) transcription factors related to lipid metabolism (SREBP1 and C/EBPβ). (**H**) *CIDEB* knockdown markedly decreased the intracellular TAG content. (**I**,**J**) The effect of *CIDEB* knockdown on lipid droplet formation. Scale bar = 400 μm. * *p* < 0.05, ** *p* < 0.01.

To further examine the role of CIDEB in GMECs, a small interfering RNA (siRNA) against CIDEB was used to knockdown its expression. The siRNA effectively reduced the mRNA (Figure 3A) and protein levels of CIDEB compared to those in control cells (Figure 3B,C). Consequently, *CIDEB* knockdown led to a significant decrease in the mRNA expression of *FASN*, *ACSS2*, *ACACA*, and *SCD1* (*p* < 0.05), but had minimal effects on the expression of *ELOVL6* (*p* > 0.05; Figure 3D). Moreover, *CIDEB* knockdown significantly decreased the expression of *DGAT1* (*p* < 0.05; Figure 3E) and *XDH* (*p* < 0.05; Figure 3F). We hypothesized that the reduced expression levels of those genes would lead to a decline in intracellular TAG content. Indeed, the interference of *CIDEB* did result in a decrease in the intracellular content of TAG, as confirmed by our experimental findings (*p* <0.05; Figure 3H). We also found that *CIDEB* knockdown markedly downregulated *SREBP1* and *CEBPB* expression (*p* <0.05; Figure 3G). Furthermore, the knockdown of CIDEB not only resulted in a reduction in the relative abundance of C16:0 and total C18:2 (Table 1), but also coupled with a significant reduction in lipid droplet accumulation in GMECs (*p* < 0.01; Figure 3I,J). Our findings indicated that *CIDEB* positively promotes fatty acid synthesis and lipid formation in GMECs. 

### 2.4. XBP1 Promotes CIDEB Transcription Activation

**Figure 4:**
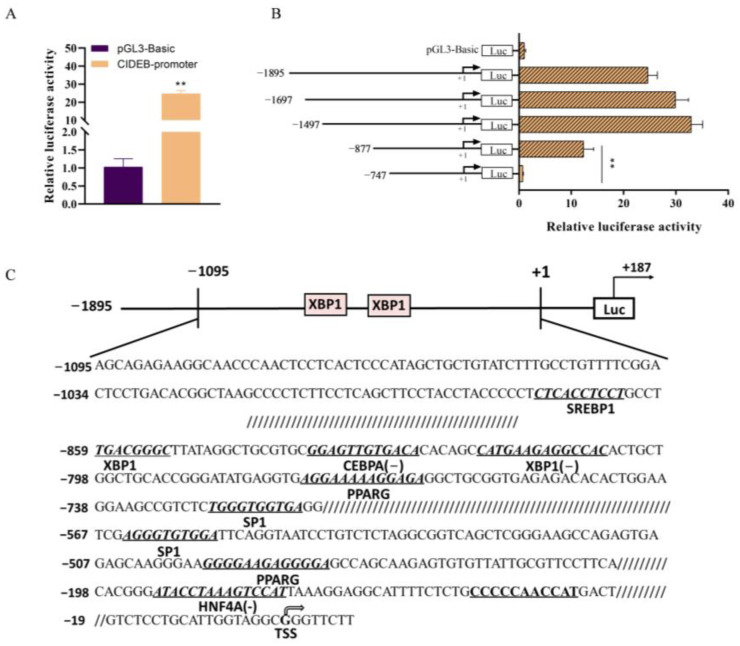
Deletion analysis and characterization of the CIDEB promoter in GMECs. (**A**) Relative luciferase activity of CIDEB promoter. (**B**) Relative luciferase activity of different lengths of CIDEB promoter. GMECs were transfected with serial ATGL promoter (−1895 bp/+187 bp, 1697 bp/+187 bp, 1497 bp/+187 bp, 877 bp/+187 bp, and 747 bp/+187 bp) for 48 h, and promoter activity was detected. ** *p* < 0.01. (**C**) Schematic representation of the goat CIDEB promoter. TSS represents transcriptional start site. The putative binding sites are indicated in boldface, and the names are shown below the underlined regions.

Gene expression is regulated through the binding of upstream transcription factors to the gene promoter. Although the functionality of CIDEB has been studied in GMECs, the regulatory mechanisms of the *CIDEB* promoter are still unclear. Thus, we cloned the full length 2082 bp *CIDEB* promoter and transfected it into GMECs for 48 h. We found that the *CIDEB* promoter activity was significantly higher than that of the pGL3-basic group (*p* < 0.05; Figure 4A), suggesting that the *CIDEB* promoter exhibits intrinsic transcriptional activity. To further explore the properties of this promoter, we generated five promoter fragments of varying lengths (−1895/+187, −1697/+187, −1497/+187, −1877/+187, −747/+187) through progressive deletion. The fragments were then subcloned into luciferase reporter vectors for further analysis. We observed a gradual increase in promoter activity as the region spanning from −1895 to −1497 was progressively deleted (Figure 4B), suggesting the presence of potential negative regulatory factors. However, when the region from −877 to −747 was deleted, the promoter activity was nearly abolished (Figure 4B), indicating the critical importance of this region in maintaining the basal activity of the promoter. 

**Figure 5:**
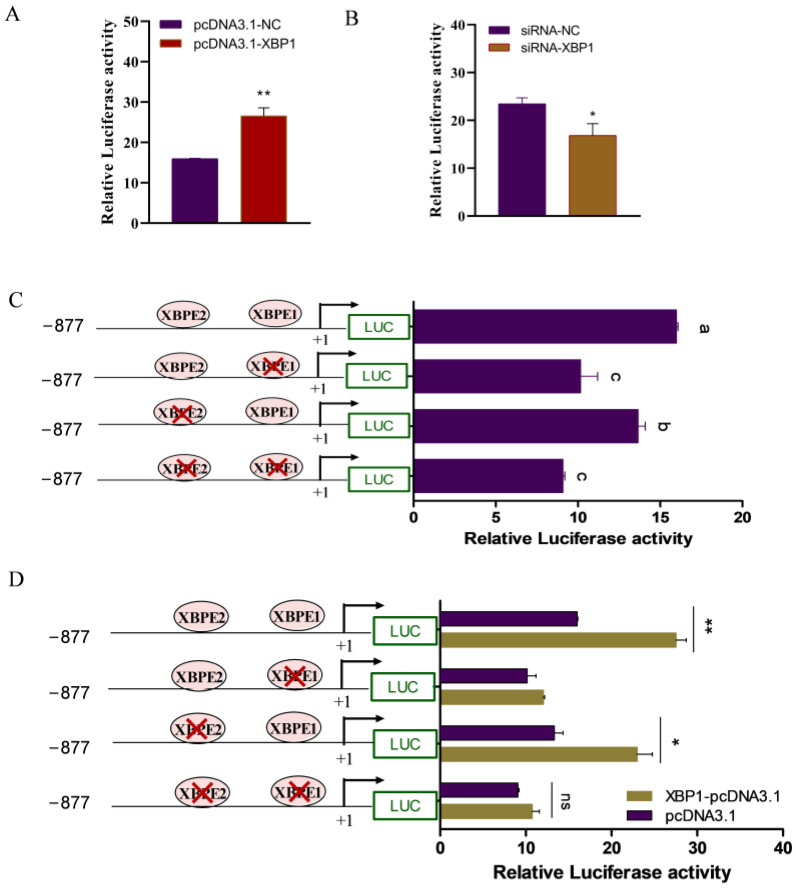
XBP1 promotes CIDEB promoter activity via XBP1 binding sites in GMECs. (**A**) XBP1 overexpression increased the full length of CIDEB promoter activity. (**B**) XBP1 knockdown decreased the CIDEB promoter activity. (**C**) The CIDEB promoter activity was decreased when the XBP1 binding sites were mutated. GMECs were co-transfected with CIDEB promoter (−877/+178) individually or simultaneously with mutated XBP1 binding site constructs and TK-Renilla for 48 h. The locations of the mutations were indicated by red crosses. Lowercase letters represent significant differences. (**D**) XBP1 promotes CIDEB promoter activity via XBP1 binding sites located in the CIDEB promoter in GMECs. Data shown are the mean ± SEM. Red cross indicates mutation sites. ns *p* > 0.05. ** *p* < 0.01. * *p* < 0.05.

Next, we performed a bioinformatics analysis of the *CIDEB* promoter using online software tools. We discovered two binding sites for *XBP1* and binding sites for transcription factors (i.e., *SERBP1*, *CEBPA*, and *PPARG*) related to lipid metabolism within the *CIDEB* promoter (Figure 4C). Thus, we next examined the effect of *XBP1* on the activity of the *CIDEB* promoter in GMECs. Subsequently, GMECs were co-transfected with constructs harboring truncated *CIDEB* promoter fragments and either XBP1-pcDNA3.1 or siXBP1. We found that overexpression of *XBP1* markedly increased *CIDEB* promoter activity (*p* < 0.01; Figure 5A), whereas *XBP1* knockdown significantly decreased *CIDEB* promoter activity (*p* < 0.05; Figure 5B), indicating that *XBP1* has a positive effect on *CIDEB* promoter activity.

### 2.5. XBP1 Promotes CIDEB Promoter Activity via XBP1 Binding Sites

As illustrated in Figure 4C, we identified two XBP1 binding sites in the CIDEB promoter. To determine which binding site is responsible for XBP1-mediated regulation of CIDEB, constructs containing XBP1 binding site (XBPE) mutants of the CIDEB promoter were generated and transfected into GMECs. We found that CIDEB promoter activity was markedly decreased when XBPE1 was individually mutated, whereas the XBPE2 mutation resulted in a more moderate reduction of approximately 10% compared to the wild type observed when XBPE2 was mutated (Figure 5C). The luciferase reporter assay showed no marked difference in promoter activity when the XBPE1 and XBPE2 sites were simultaneously mutated compared to that in the vector with the XBPE1 mutation alone (Figure 5C). Furthermore, overexpression of XBP1 significantly increased the transcriptional activity of the CIDEB promoter when XBPE2 was mutated individually. Intriguingly, the stimulatory effect of XBP1 was almost abrogated when both the XBPE1 and XBPE2 binding elements were mutated simultaneously (Figure 5D). Collectively, our findings suggested that XBP1 promotes CIDEB promoter activity through its interaction with the XBP binding elements present within the regulatory region of the CIDEB gene in GMECs.

### 2.6. XBP1-Mediated Modulation of CIDEB Altered Lipid Metabolism Processes

**Figure 6:**
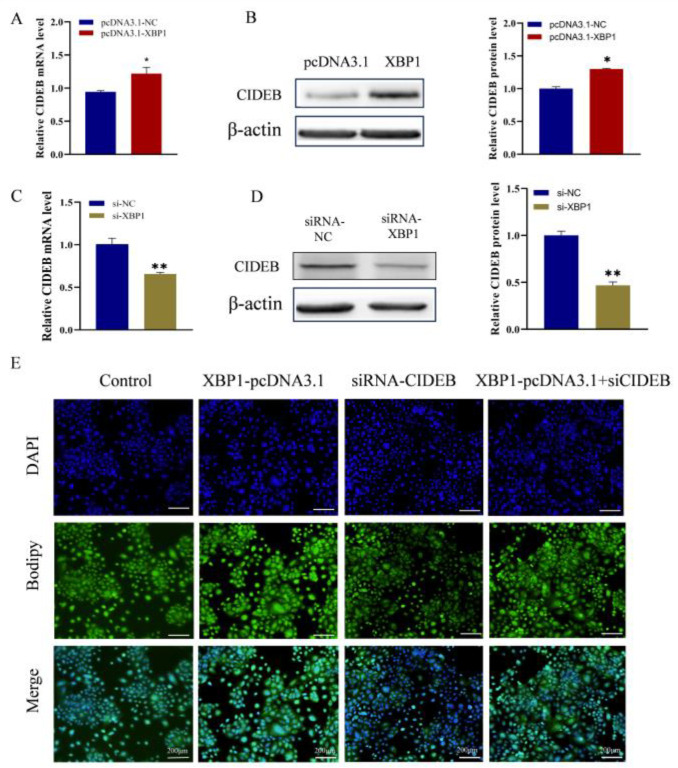
XBP1 promotes fatty acid synthesis by regulating CIDEB expression in GMECs. (**A**,**B**) The mRNA and protein levels of CIDEB after transfected with XBP1-pcDNA3.1. (**C**,**D**) The mRNA and protein levels of CIDEB after transfected with siRNA-XBP1. (**E**) Lipid droplets in GMECs were detected by BODIPY 493/503 staining. Scale bar = 200 μm. ** *p* < 0.01. * *p* < 0.05.

To our knowledge, XBP1 has been identified as a pivotal transcription factor that plays a key role in regulating cellular endoplasmic reticulum (ER) stress and lipid metabolism [21]. To further explore the interaction between XBP1 and CIDEB, GMECs were co-transfected into either XBP1-pcDNA3.1or siRNA-XBP1, and then TAG or lipid droplets were detected. We found that pcDNA3.1-XBP1 effectively increased CIDEB mRNA levels (*p* < 0.05; Figure 6A), resulting in the substantial upregulation of the CIDEB protein relative to that in the control cells (*p* < 0.05; Figure 6B). In contrast, XBP1 knockdown markedly decreased the expression of the CIDEB at both the mRNA and protein levels (Figure 6C,D). Furthermore, we observed a marked increase in lipid droplet levels in cells overexpressing XBP1, but a notable decrease in cells deficient in CIDEB. As predicted, interference of CIDEB counteracted the promoting effect of XBP1 on lipid droplet formation (Figure 6E). Therefore, we hypothesized that XBP1 modulated fatty acid synthesis by regulating CIDEB expression.

## 3. Discussion

Milk fat is the indicator of milk’s nutritional value, and the synthesis of fatty acid is of great importance in goat mammary glands. Particularly, goat milk is rich in short- and medium-chain fatty acids and unsaturated fatty acids, which are beneficial to the human body [22]. Some studies have indicated that the quality and health-promoting characteristics of goat milk are closely related to their fatty acid content [23]. CIDEB is a protein associated with the endoplasmic reticulum and lipid droplets, and it is involved in regulating lipid metabolism and related diseases, thus serving as an important modulator of lipid homeostasis [24]. In this study, enrichment of H3K4me3 and H3K27ac within the CIDEB promoter region was substantially higher at peak lactation compared to the dry period. Notably, CIDEB overexpression promoted fatty acid synthesis and lipid droplet accumulation, suggesting that CIDEB may play an important role in regulating lipid metabolism in GMECs.

CIDEB is an important factor involved in the regulation of processes such as lipid synthesis, lipid droplet formation, and degradation, thereby preserving the overall homeostatic balance of cellular lipid levels [25]. In the liver, knockout of CIDEB in mice leads to a significant decrease in body weight and hepatic fat synthesis rate [8]. Additionally, in the small intestine, CIDEB interacts with ApoB48 to promote lipidation and chylomicron assembly, thereby facilitating lipid secretion [26]. In contrast, CIDEB inhibits lipid accumulation and regulates lipid metabolism by controlling the storage and release of fats from renal tubular epithelial cells [27]. In this study, we found that CIDEB overexpression significantly upregulated genes related to fatty acid and TAG synthesis. This was further confirmed by the increased lipid droplet accumulation. However, the specific effects of CIDEB on fatty acid metabolism may vary depending on the animal model or tissue studied, suggesting diverse regulatory roles of CIDEB in lipid homeostasis that warrant further investigation.

Previous studies reported that CIDEB knockout in mice reduces the activation of SREBP, thereby affecting its regulation of genes involved in the de novo synthesis of cholesterol and fatty acids [9]. In the current study, the inhibition of CIDEB expression significantly reduced the mRNA level of SREBP1. SREBP1 regulates fatty acids and TAG synthesis by regulating the expression of *ACACA*, *FASN*, and *SCD1* [28,29], which synergistically promote de novo synthesis of fatty acids using acetyl-CoA. In this study, overexpression of the CIDEB gene significantly increased the *ACACA* expression, while *CIDEB* knockdown significantly inhibited *FASN*, *ACACA*, and *SCD1* expression. This suggests that *CIDEB* affects fatty acid synthesis by regulating the coordinated expression of *ACACA* and *FASN*. Furthermore, although *CIDEB* knockdown increased the percentage of C16:0, the expression of *ELOVL6* (an enzyme involved in the elongation of fatty acids with carbon chain lengths of C12 to C16 [30]) remained unchanged. This may help to preserve lipid homeostasis during declines in C16:0 levels.

In bovine mammary epithelial cells, XDH has a critical regulatory function in milk synthesis and secretion by promoting the secretion of milk lipid droplets [31]. In our study, CIDEB overexpression significantly upregulated XDH expression, leading to the accumulation of lipid droplets. This indicates that CIDEB alters the expression of lipid droplet-related genes, thereby further affecting the lipid droplet accumulation. Additionally, a previous study showed that CIDEB knockout resulted in decreased plasma TAGs and non-esterified fatty acid levels [8], which is consistent with our findings. Furthermore, inhibition of FASN and ACACA leads to apoptosis in cancer cells, whereas the addition of exogenous fatty acids restored normal cell growth [32]. Research has shown that the overexpression of *CIDEB* promotes cell apoptosis [33]. However, whether CIDEB affects GMECs apoptosis in GMECs requires further investigation. 

Previous study has shown that SP1, SP3, and HNF-4α are key regulatory factors for the basal transcriptional activity of human CIDEB promoters [34]. In the present study, we found that the goat CIDEB promoter region also contains multiple binding sites for SP1, SP3, and HNF-4α, suggesting that these transcription factors play an important role in the transcriptional regulation of the goat CIDEB gene. Moreover, the production of hepatitis B virus may lead to the downregulation of HNF4α expression, which in turn results in the impairment of CIDEB gene expression [35]. Previous findings have shown that C/EBPα is able to bind to the active regions of the PPARG promoter and promote its transcription [36]. We also found potential binding sites for C/EBPα and PPARG on the CIDEB promoter region, suggesting that C/EBPα and PPARG may interact with each other and jointly influence the activity of CIDEB promoter.

XBP1 is a key transcriptional regulator that integrates cellular responses to ER stress by regulating lipid metabolism and homeostasis [13,37]. Previously studies showed that *XBP1* directly binds to the promoter of acyl-CoA oxidase 1 (ACOX1) and peroxisome proliferator-activated receptor α (PPARA), stimulating their expression to promote hepatic lipogenesis [38,39]. XBP1 can also activate the expression of fibroblast growth factor 21 (FGF21), which in turn enhances the activity of PPARG, thereby regulating the transcription of various genes involved in fatty acid transport and metabolism [40]. In the current study, *XBP1* overexpression strongly upregulated CIDEB expression and markedly increased the lipid droplet accumulation in GMECs. Moreover, knockdown of CIDEB was shown to attenuate the impact of *XBP1* on lipid droplet and TAG contents. Although two XBP1 binding sites are found on the CIDEB promoter, the stimulatory effect of XBP1 on CIDEB persists even when the XBPE2 site is mutated, suggesting that *XBP1* indirectly binds to the XBPE2 site. Notably, mutation of the XBPE1 and XBPE2 sites on the CIDEB promoter almost abrogated the stimulatory impact of *XBP1* on CIDEB promoter activity. These findings suggest that XBPE is required for XBP1 to regulate the transcription of CIDEB.

## 4. Materials and Methods

### 4.1. Chromatin Immunoprecipitation and Sequencing (ChIP-Seq)

The goat mammary gland tissues at early lactation, peak lactation, mid lactation, and the dry period were stored in our laboratory. Initially, the samples were blended in a mortar with a grinding bar while cooled with liquid nitrogen. Throughout the grinding, any material that adhered to the pestle was carefully scraped back into the mortar using a small spoon. The samples were fixed using a final concentration of 1% formaldehyde at room temperature (RT) for 10 min. To stop the fixation process, 0.125 M glycine was added, and the samples were then centrifuged at 3000 rpm for 5 min. Then, the cells were lysed using 300 μL of SDS lysis buffer (P0013G, Beyotime, Shanghai, China) containing 1x protease and the DNA were sheared using a Bioruptor sonication system (Bioruptor Pico, Diagenode, Liège, Belgium) with the following program: 30 s ON, 30 s OFF, for 10–12 cycles. For the immunoprecipitation (IP) experiments, H3K27ac (ab4729, Abcam, Cambridge, MA, USA), anti-H3K4me3 (ab8580, Abcam, Cambridge, MA, USA), and IgG antibody (ab171870, Abcam, Cambridge, MA, USA) were used as the primary antibodies. Following the IP, the DNA was purified using the PCI method (phenol-chloroform-isoamyl alcohol). The ChIP-seq data were aligned to a reference genome using Bowtie 2 and histone deposition sites were identified using MACS2. To visualize the enrichment of peaks on genes, the Integrated Genomics Viewer (IGV; v2.11.1) software was utilized.

### 4.2. Cell Culture

GMECs were isolated from five 3-year-old Xinong Sannen dairy goats during peak lactation period (60 d after parturition), the nutritional level suitable for the lactation period of dairy goats, following the previously described method [41]. Detailed protocols for purification and authentication can be found in earlier studies [42,43]. Briefly, mammary gland tissue (1 mm cubes) were plated in 60 mm dishes, which incubated at 37 °C with 5% CO_2_ until the cells detached from the tissue block. Then, the cells were cultured in growth medium comprised 90% basal DMEM/F12 (10-092-CVRC, New York, NY, USA) medium, 5 mg/L bovine insulin (16634, Sigma, St. Louis, MO, USA), 5 μg/mL hydrocortisone (H0888, Sigma), 100 U/mL penicillin/streptomycin (080092569, Harbin, China), 10 ng/mL epidermal growth factor (PHG0311, Invitrogen, Waltham, MA, USA), and 10% fetal bovine serum (Hyclone, Waltham, MA, USA). To induce lactogenesis, the cells were incubated in lactation medium supplemented with prolactin (L6520, 2.5 μg/mL, Sigma) for 48 h before proceeding with subsequent experiments.

### 4.3. Reverse Transcription Real-Time PCR (RT-qPCR)

GMECs were cultured overnight in 12-well plates and then transfected with a CIDEB overexpression vector or siRNA for 48 h. Total RNA was extracted from both the GMECs and mammary gland tissues using RNAiso Plus reagent (9109, Takara, Kusatsu, Japan) according to the manufacturer’s instructions. The RNA concentration was measured using a spectrophotometer (Nanodrop 2000, Thermo, Rockford, IL, USA), and 500 ng of total RNA was used for cDNA synthesis with the PrimeScript RT Reagent Kit (Perfect Real Time, Takara). The relative expression values were normalized to the levels of the ubiquitously expressed transcript (UXT) and ribosomal protein S9 (RPS9) and calculated using the 2^−ΔΔCt^ method. Each sample was analyzed in three biological replicates. The primer sequences for the target genes can be found in Table 2.

### 4.4. Plasmid Construction

Primers containing specific restriction sites were designed based on the predicted sequence of CIDEB and XBP1 from NCBI, as well as considering the multiple cloning sites of the pcDNA3.1 vector. Then, the complete CIDEB and XBP1 sequences were cloned from the cDNA of goat mammary gland tissue and subcloned into the expression vector. The sequences of primer were as follows: CIDEB-F: CGCGGATCCATGGAGTACCTCTCTAACC; CIDEB-R: GCTCTAGATCAGTAGGGTTTAAGGCGA; XBP1-F: CGCGGATCCATGGTGGTGGTTGCACCCGC; XBP1-R: AAGCGGCCGCTTAGACACTAATCAGCTGGG.

The 2082 bp fragment of PPARGC1A promoter was obtained from goat genomic DNA using PrimeSTAR HS DNA polymerase (Takara Bio Inc., Kusatsu, Japan). Various deletion fragments were utilized by designing primers at specific positions upstream of the transcription start site (1895 bp, 1697 bp, 1497 bp, 877 bp, 747 bp), with an additional 187 bp downstream of the transcription start site. The DNA fragments containing different lengths were then subcloned into the pGL3-basic vector, which were digested with KpnI and HindIII. Overlapping extended PCR was used to introduced site-directed mutations in XBPE regions as previously described [44]. The http://gene-regulation.com/ and http://jaspar2014.genereg.net/ (accessed on 24 August 2023) was used to predict the transcription factor binding sites in the CIDEB promoter. The specific primers utilized in these experiments are listed in Table 3.

### 4.5. BODIPY Staining

GMECs were seeded into 12-well plates and transfected with either overexpression plasmids or siRNA. After 48 h, the GMECs were washed three times with PBS and fixed with 4% paraformaldehyde at 4 °C for 30 min. To stain the lipid droplets, a 0.1% BODIPY 493/503 solution (D3922, Invitrogen) was added and incubated for 30 min. Subsequently, the cells were counterstained with DAPI solution (C1006, Beyotime, Shanghai, China) for 5 min to visualize the cell nuclei. After staining, the cells were washed three times with PBS. The images of the lipid droplets were captured using a cell imaging reader (BioTek Instruments Inc, Winooski, VT, USA). The fluorescence intensity of BODIPY was used to indicate the content of lipid droplets, which was normalized by DAPI staining.

### 4.6. Cellular TAG and Cholesterol Analysis

GMECs were seeded into 6-well plates and transfected with either overexpression plasmids or siRNA for 48 h. Then, the cells were lysed for 10 min on ice. GPO-Trinder triacylglycerol assay kits (E1013, Applypen Technologies, Beijing, China) and cholesterol (E1015, Applygen Technologies) assay kits were used to detect cellular TAG and cholesterol detection on a Biotek microplate reader (Winooski, VT, USA). TAG and cholesterol concentration was corrected by protein concentration (µg/mg protein), which were detected by the BCA protein assay kit (23227, Thermo Fisher Scientific, Rockford, IL, USA).

### 4.7. Fatty Acids Extraction and Analysis

GMECs were seeded in 60 mm plates and transfected with siRNA with a confluency of 70% to 80% for 48 h. Then, the cells were washed three times with pre-cold PBS. A total of 2 mL of 2.5% methanol-sulfuric acid solution was added to the culture dish and then all the cells were transferred to an 8 mL microcentrifuge tube and sonicated for 10 min. Subsequently, 2 mL 0.1 M HCL and 800 μL of n-hexane was added and centrifuged at 3000 rpm for 5 min. Finally, 200 μL of lipid reconstituted solution was extracted and analyzed by UPLC-MS/MS (UPLC, Shim-pack UFLC SHIMADZU CBM30A; MS, Applied Biosystems SCIEX 6500+QTRAP, Waltham, MA, USA).

### 4.8. Protein Extraction and Western Blotting

GMECs were cultured in six-well plates and transfected with either overexpression plasmids or siRNA for 48 h. After washing the cells with PBS buffer, they were lysed in RIPA lysis buffer (R0010, Solarbio, Beijing, China) supplemented with cOmplete Protease Inhibitor Cocktail (04693132001, Roche Diagnostics Ltd., Mannheim, Germany). The protein concentration was determined by BCA assay kit (#23225, Thermo Fisher Scientific, USA).

Equal amounts of total protein (20 μg per lane) were separated on a 10% SDS-PAGE gel. The proteins were then transferred onto PVDF membranes and blocked with 5% skim milk (232100, BD, Franklin Lakes, NJ, USA). For protein detection, CIDEB rabbit polyclonal antibody (27600-1-AP, Proteintech, Wuhan, China, 1:1000) and β-actin mouse monoclonal antibody (CW0096, CW Biotech, Beijing, China, 1:1000) were used and incubated overnight at 4 °C. Goat anti-rabbit (CW0103, CW Biotech, 1:2000) and goat anti-mouse (CW0102, CW Biotech, 1:2000) HRP-conjugated IgG were used as secondary antibodies. Protein signals were visualized using a chemiluminescent (ECL) western blot system (1705061, Bio-Rad, Hercules, CA, USA).

### 4.9. Cell Transfection and Luciferase Assay

GMECs were cultured in 48-well plates and transfected with 300 ng CIDEB promoter plasmids or co-transfected with XBP1-pcDNA3.1/siXBP1 and CIDEB promoter using X-tremeGENE HP DNA transfection reagent (Roche, Germany) according to the manufacturer’s protocol. For internal control, the Renilla luciferase vector was co-transfected with the pGL3-CIDEB promoter. The ratio of pRL-TK to the pGL3-CIDEB promoter used for co-transfection was 1:50. After transfection for 48 h, the cells were washed three times with pre-cold PBS and the promoter was examined by the dual-luciferase reporter assay system (E1910, Promega, Madison, WI, USA). 

### 4.10. Statistical Analysis

The data presented in this study are expressed as mean ± SEM, and all experiments were conducted in triplicate to ensure reproducibility. Statistical analysis was performed with Student’s *t*-test for only two groups and one-way ANOVA was performed with Duncan’s test for multiple comparisons. The statistical analysis was performed using SPSS 20.0, and the significance level was set at * *p* < 0.05 and ** *p* < 0.01, indicating statistical significance.

## 5. Conclusions

**Figure 7:**
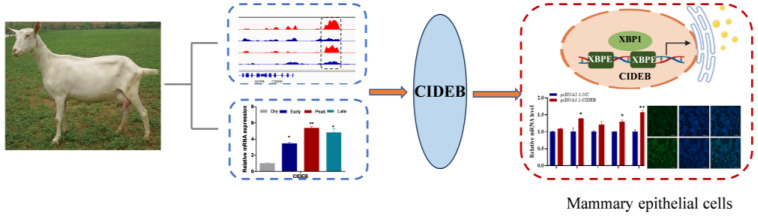
We identified a novel active lactation gene CIDEB using ChIP-seq from goat mammary gland at different lactation stages, and its expression was significantly elevated during the peak lactation compared to the dry period, suggesting that CIDEB plays an important role in adapting to the high metabolic demands associated with lactation. Firstly, CIDEB promotes milk fatty acid and triglyceride synthesis in goat mammary epithelial cells (GMECs). Furthermore, XBP1 promotes CIDEB transcription via XBP1 binding sites located on the CIDEB promoter, leading to increased lipid droplet accumulation in GMECs. Our data revealed the stimulatory mechanism of CIDEB in fatty acid metabolism in dairy goats. ** *p* < 0.01. * *p* < 0.05.

In conclusion, our study revealed that CIDEB is a critical regulator of fatty acid synthesis and lipid droplet formation in GMECs, thus providing insight into the molecular mechanisms underlying milk composition in dairy goats. Importantly, our findings also revealed that transcriptional regulation of CIDEB by XBP1 is mediated via XBP1 binding sites in GMECs (Figure 7). Although this is the first study to reveal the mechanism of CIDEB in fatty acid synthesis, certain limitations in this study should be noted. For example, ChIP assays could be employed to elucidate the effect of XBP1 on the CIDEB promoter. Moreover, additional studies should be performed to elucidate the mechanism by which XBP1 regulates CIDEB expression during lactation in dairy goats.

## Figures and Tables

**Table 1:** Effect of interference of CIDEB on fatty acid composition in GMECs.

Fatty Acid (%)	si-NC	si-*CIDEB*
C6:0	16.02 ± 0.72	17.51 ± 0.55
C8:0	3.12 ± 0.17	3.62 ± 0.30
C10:0	1.93 ± 0.20	2.14 ± 0.25
C11:0	1.39 ± 0.04	1.50 ± 0.07
C12:0	1.48 ± 0.06	2.05 ± 0.04
C14:1	15.65 ± 0.35	15.02 ± 0.23
C15:0	1.56 ± 0.05	1.67 ± 0.01
C16:0	7.16 ± 0.42 ^a^	6.24 ± 0.08 ^b^
C18:0	11.14 ± 0.21	11.66 ± 0.22
C18:1	25.42 ± 0.60	24.85 ± 1.23
C18:2	4.55 ± 0.04 ^a^	4.35 ± 0.08 ^b^
C22:1	10.06 ± 0.50	9.37 ± 0.31

Fatty acid data are shown as a ratio of the total fatty acids. Data are presented as mean ± SEM. Lowercase letters represent significant differences.

**Table 2:** Real time quantitative PCR primers.

Gene		Primer Sequence (5′-3′)	Size (bp)	Temperature (°C)
*UXT*	F R	CAGCTGGCCAAATACCTTCAA GTGTCTGGGACCACTGTGTCAA	125	60
*RPS9*	F R	CCTCGACCAAGAGCTGAAG CCTCCAGACCTCACGTTTGTTC	64	60
*CIDEB*	F R	CTCAGGTCAGTATCCAACAT GAGCCTTGTCTAGCAGTT	149	60
*FASN*	F R	GGGCTCCACCACCGTGTTCCA GCTCTGCTGGGCCTGCAGCTG	226	60
*ACACA*	F R	CTCCAACCTCAACCACTACGG GGGGAATCACAGAAGCAGCC	171	60
*ACSS2*	F R	GGCGAATGCCTCTACTGCTT GGCCAATCTTTTCTCTAATCTGCTT	100	60
*SCD1*	F R	CCATCGCCTGTGGAGTCAC GTCGGATAAATCTAGCGTAGCA	257	60
*ELOVL6*	F R	GGAAGCCTTTAGTGCTCTGGTC ATTGTATCTCCTAGTTCGGGTGC	205	60
*XDH*	F R	GATCATCCACTTTTCTGCCAATG CCTCGTCTTGGTGCTTCCAA	100	60
*PLIN2*	F R	GATGAGACCACGGCAGATG GTCAACTATTTCCCGCACAAG	120	60
*DGAT1*	F R	CCACTGGGACCTGAGGTGTC GCATCACCACACACCAATTCA	111	60
*GPAM*	F R	ATTGACCCTTGGCACGATAG AACAGCACCTTCCCACAAAG	188	60
*AGPAT6*	F R	AAGCAAGTTGCCCATCCTCA AAACTGTGGCTCCAATTTCGA	101	60
*SREBP1*	F R	ACGCCATCGAGAAACGCTAC GTGCGCAGACTCAGGTTCTC	181	60
*LXRα*	F R	CATCAACCCCATCTTCGAGTT CAGGGCCTCCACATATGTGT	163	60
*C/EBPβ*	F R	GCCTGTCCACGTCCTCGTCGTCCAGC CGGATCTTGTACTCGTCGCTGTGCTTGTCC	169	60
*XBP1*	F R	AGTTAAGACAGCGGTTGGGG CACTCCATTCCCCTTGGTCT	71	60

**Table 3:** Primers for deleted and site-directed Mutant for CIDEB Promoter.

Name	Sequence (5′-3′)	Binding Region
PF1	*GG*GGTACCACATTCCTCTGTCCTTCCG	−1895
PF2	*GG*GGTACCAACCCATATCCCCTGCTGTC	−1697
PF3	*GG*GGTACCTTGGGGGCTCTTCAGGGCCC	−1497
PF4	*GG*GGTACCGGAGATGCACGGAGGTGTGA	−877
PF5	*GG*GGTACCACACTGGAAGGAAGCCGTCT	−747
*XBP1* mut1	GACACACAGCACTGCTGGGC	−805
*XBP1* anti-mut1	GCCCAGCAGTGCTGTGTGTC	−805
*XBP1* mut2	CGGAGGTGTAATGTGATTATAGGCT	−861
*XBP1* anti-mut2	GTAATGTGATTATAGGCTGCGTGCGGAG	−861
PR	*CCC*AAGCTTCTTCCCTGCTCTTCCTTCT	+187

“+” and “−” represent upstream and downstream from the transcriptional start site; restriction enzyme sites were labelled by bold.

## Data Availability

The data supporting the findings of this study are available within the article. For any further inquiries, contact the corresponding author.
